# Hospital–Medicare Advantage Vertical Integration and Cardiopulmonary Care in Integrated Hospitals

**DOI:** 10.1001/jamahealthforum.2025.5648

**Published:** 2025-12-26

**Authors:** Geronimo Bejarano, Meehir N. Dixit, Alexander P. Philips, Karen E. Joynt Maddox, Meredith B. Rosenthal, Amal Trivedi, David J. Meyers

**Affiliations:** 1 Department of Health Services, Policy, and Practice, Brown University School of Public Health, Providence, Rhode Island; 2 Cardiovascular Division, John T. Milliken Department of Internal Medicine, Washington University School of Medicine in St Louis, St Louis, Missouri; 3 Department of Health Policy & Management, Harvard T.H. Chan School of Public Health, Boston, Massachusetts; 4 Providence Veterans Affairs Medical Center, Providence, Rhode Island

## Abstract

**Importance:**

The rate of health care payer and practitioner consolidation is increasing, with limited assessment of hospital–Medicare Advantage (MA) plan vertical integration. Little is known about whether aligned incentives between hospitals and MA contracts may improve care coordination and outcomes.

**Objective:**

To investigate the association between hospital–MA contract vertical integration and cardiopulmonary outcomes.

**Design, Setting, and Participants:**

This cross-sectional study included MA enrollees admitted for acute myocardial infarction, heart failure, or pneumonia between January 2015 and December 2022. Admissions that occurred in a hospital that owns an MA plan were matched to comparison admissions based on the propensity score of being enrolled in an integrated MA contract. Analysis was conducted between December 1, 2024, and March 20, 2025.

**Exposure:**

Admissions were categorized as nonintegrated (MA contract not owned by a hospital), partially integrated (hospital-owned MA contract but admission at nonaffiliated hospital), or fully integrated (admitted at a hospital that owns MA contract).

**Main Outcomes and Measures:**

Generalized linear models adjusted for demographics, clinical features, and hospital, zip code, year, and diagnosis-related group fixed effects. Care intensity was measured using length of stay and intensive care unit (ICU) use. Clinical outcomes were inpatient mortality, 30-day postdischarge mortality, and 30-day postdischarge readmission.

**Results:**

The sample consisted of 1 057 715 admissions, of which 234 587 were partially integrated and 118 017 were fully integrated. After matching, the mean (SD) age was 78.0 (9.9) years for participants in the nonintegrated group, 78.0 (9.9) years for those in the partially integrated group, and 78.0 (9.8) years in the fully integrated group; 48.8% were female in the nonintegrated group, 48.9% in the partially integrated group, and 48.9% in the fully integrated group. Fully integrated admissions had significantly shorter lengths of stay (adjusted difference, −0.24 days; 95% CI, −0.31 to −0.18 days vs nonintegrated; −0.28 days; 95% CI, −0.37 to −0.18 days vs partially integrated) and lower rates of ICU use (−1.17 percentage points; 95% CI, −1.82 to −0.52 percentage points vs nonintegrated; −1.42 percentage points; 95% CI, −2.44 to −0.40 percentage points vs partially integrated), inpatient mortality (−0.61 percentage points; 95% CI, −0.88 to −0.34 percentage points vs nonintegrated), 30-day postdischarge mortality (−0.87 percentage points; 95% CI, −1.30 to −0.44 percentage points vs nonintegrated), and 30-day postdischarge readmission (−0.78 percentage points; 95% CI, −1.30 to −0.26 percentage points vs nonintegrated).

**Conclusions and Relevance:**

In this study, among patients hospitalized with myocardial infarction, heart failure, or pneumonia, enrollment in a hospital-owned MA contract along with admission to the affiliated hospital was associated with a shorter length of stay, less ICU use, and lower mortality and readmission rates.

## Key Points

**Question:**

Is hospital–Medicare Advantage plan vertical integration associated with cardiopulmonary outcomes?

**Findings:**

In this cross-sectional study including 1 057 715 admissions, fully compared with nonintegrated admissions had shorter lengths of stay (−0.24 days) and lower rates of any intensive care unit use (−1.17 percentage points), inpatient mortality (−0.61 percentage points), 30-day postdischarge mortality (−0.87 percentage points), and 30-day postdischarge readmission (−0.78 percentage points). Fully compared with partially integrated admissions had shorter lengths of stay (−0.28 days) and lower rates of any intensive care unit use (−1.42 percentage points).

**Meaning:**

These findings suggest potential benefits from aligning incentives between insurers and hospitals.

## Teaser

This cross-sectional study examines associations between level of hospital-Medicare Advantage (MA) vertical integration exposure with care intensity and clinical outcomes among MA enrollees hospitalized for cardiopulmonary disease.

## Introduction

Health care payers and practitioners in the US are increasingly horizontally and vertically integrated.^1,2,3^ Horizontal integration occurs when organizations at the same level of the supply chain merge.^4^ Vertical integration occurs when 2 organizations at different levels of the supply chain merge, such as when a hospital or insurer acquires a physician group or a hospital acquires an insurer.^3,4^ Current evidence suggests that horizontal integration, mainly hospital mergers, is associated with increased health care costs without improvement in patient outcomes.^5,6,7^ Similarly, vertical integration between hospital and physician groups has been associated with higher costs and no meaningful improvement in health outcomes.^8,9,10^ Little is known, however, about the association of hospital-insurer vertical integration with patient outcomes.^11^

For a variety of reasons, including revenue diversification, an increasing number of hospitals have vertically integrated with insurers, most prominently through Medicare Advantage (MA) contracts.^11,12,13,14^ Hospitals either partner with an existing MA insurer to create a new MA contract through a joint venture or create their own MA contract. These hospital-owned MA contracts are new MA contracts, not previously existing MA contracts that are acquired by the hospital.^11,13^ Enrollment in hospital-owned MA contracts had increased to almost 1 in every 6 MA beneficiaries as of 2020.^11^ Proponents of vertical integration in health care point to the possibility of enhanced coordination and aligned incentives between practitioners and insurance companies, which may improve care efficiency and patient outcomes.^4,15,16,17,18,19^ Common ownership between the hospital and MA contract incentivizes the hospital to reduce unnecessary spending from its beneficiaries, including spending on elective procedures, readmissions, and care delivered at other nonaffiliated health care sites. Additionally, there is an increase in the amount and type of data that the hospital and MA contract have access to after vertical integration.^20^ If used appropriately, the improved data (eg, hospital access to nonhospital care and pharmaceutical claims) can provide opportunities for quality improvement efforts to streamline and further individualize care for beneficiaries.^20,21^

However, critics have raised concerns that hospital-MA vertical integration will lead to increasing health care costs without commensurate health improvements, consistent with observations in hospital-physician vertical integration and hospital-hospital horizontal integration.^6,10^ Moreover, if there is a reduction in spending after hospital-MA vertical integration, savings might not accrue to beneficiaries or the Medicare program but instead yield increased profits for the MA contract.^3,22^ Additionally, common ownership between the hospital and MA contract may allow for increased influence from the insurer on the practitioner’s coding intensity, which could raise health care costs for the Medicare program.^22,23^ Importantly, the aforementioned benefits of hospital-MA vertical integration rely on the beneficiary being admitted to an affiliated hospital. Finally, hospital-MA vertical integration may allow for anticompetitive negotiations with nonaffiliated hospitals or MA contracts that lead to termination of nonaffiliated MA contracts in that market.^24,25,26,27^

Cardiopulmonary disease provides an ideal condition by which to examine hospital-MA vertical integration as it represents a large proportion of high-intensity high-cost hospitalizations.^28,29^ Therefore, in this study, we examined the association between level of hospital-MA vertical integration exposure with care intensity and clinical outcomes among MA enrollees hospitalized with acute myocardial infarction, heart failure, or pneumonia.

## Methods

This cross-sectional study was reported in concordance with the Strengthening the Reporting of Observational Studies in Epidemiology (STROBE) reporting guideline, and review and informed consent were waived due to the use of deidentified data by the Brown University Institutional Review Board.^30^

### Data Sources and Vertical Integration Exposure

The Medicare Provider Analysis and Review dataset was used to identify MA beneficiaries who were admitted to a hospital between 2015 and 2022 with a primary diagnosis of acute myocardial infarction, heart failure, or pneumonia. Analysis was conducted between December 1, 2024, and March 20, 2025. In eTable 1 in Supplement 1, we provide the *International Statistical Classification of Diseases, Tenth Revision* codes that were used to identify these cardiopulmonary admissions. Additionally, we used the Medicare Provider Analysis and Review dataset to identify admission type (elective vs nonelective) and Elixhauser comorbidity count.^31^ The Medicare Master Beneficiary Summary File was used to determine MA enrollment and demographic characteristics. To identify hospital-MA vertical integration, we used a previously described dataset.^11,32^ Briefly, Agency for Healthcare Research and Quality compendium files were used to identify hospital-MA vertical integration. Next, we used public MA contract datasets to identify the MA contract number and date of vertical integration. We verified the hospital-MA vertical integration using industry reports and hospital and MA contract websites. Finally, we used the Agency for Healthcare Research and Quality dataset for health system to hospital merging (ie, health system to hospital cross-walk data showing which hospitals are a part of which health system) to determine the hospital identifier and create the final dataset, which includes the hospital identifier, MA contract, and date of vertical integration.

We classified admissions by level of exposure to hospital-MA vertical integration. First, we excluded those enrolled in traditional Medicare to reduce bias from favorable selection in MA compared with traditional Medicare.^33,34^ Admissions were categorized as not enrolled in a hospital-owned MA contract (nonintegrated), hospital-owned MA contract enrollee admitted at a hospital that does own an MA contract but does not own the admission MA contract (partially integrated), or hospital-owned MA contract enrollee admitted at the hospital that owns the admission MA contract (fully integrated). Due to possible differences between admissions in the level of exposure to hospital-MA vertical integration, we used a multistep propensity score–matching approach to balance the demographic and clinical characteristics (eFigure in Supplement 1). First, we required an exact match on hospital. Second, we matched 2:1 partially integrated admissions to fully integrated admissions using the propensity score likelihood of being fully integrated based on age, sex, race and ethnicity (race and ethnicity were determined according to self-report), dual-eligibility status, cardiopulmonary condition type, and Elixhauser comorbidity count. Third, we matched 2:1 nonintegrated admissions to hospital-owned MA contract admissions (both partially and fully integrated admissions) using the propensity score likelihood of being enrolled in a hospital-owned plan based on the same aforementioned covariates. The match rate was greater than 99% for both steps and created a final sample of fully integrated admissions and matched controls of partially integrated and nonintegrated admissions.

### Outcomes

Care intensity measures were length of stay and whether there was any intensive care unit (ICU) use. Length of stay was the number of days from admission to discharge; those who were discharged on the same day as admission were considered to have a length of stay of 1 day. ICU use was a binary indicator of spending at least 1 day in the ICU. Clinical outcomes were inpatient mortality, 30-day postdischarge mortality, and 30-day postdischarge readmission rates. Inpatient mortality was defined as any death during the hospital stay.

### Statistical Analysis

Summary statistics were used to describe patient age, race and ethnicity, sex, dual-eligibility status, cardiopulmonary condition type, and Elixhauser comorbidity count. We compared the association between level of vertical integration and each outcome variable. In our analysis, we estimated marginal effects using generalized linear models (GLMs) for each outcome, adjusting for age, race, sex, dual-eligibility status, and cardiopulmonary condition type, including hospital, patient zip code, year, and diagnosis-related group fixed effects. Since fixed effects were included, we compared patients within the same hospital and zip code with the same diagnosis-related group in the same year. This model allowed us to control for non–time-varying differences between hospitals, geographic locations, demographics, and diagnosis-related groups. The interpretation of these findings compares fully integrated admissions with nonintegrated and partially integrated admissions at hospitals that own an MA contract. A Poisson GLM was used for the count outcome (length of stay), and a logit GLM was used for binary outcomes (inpatient mortality, 30-day postdischarge mortality and readmission, and any ICU use). We conducted subgroup analyses stratifying by the 3 cardiopulmonary diseases. All models clustered SEs at the hospital level.

Several sensitivity analyses to test the robustness of our results were conducted. First, we checked for overdispersion in the Poisson models using the χ^2^ goodness-of-fit test (eTable 2 in Supplement 1). To mitigate overdispersion concerns, we used hospital-level clustered SEs. Second, due to potential issues with fixed effects in nonlinear models, we estimated the primary analysis using linear regression. Third, we estimated the primary model with an additional regression adjustment of Elixhauser comorbidity count in an attempt to reduce any residual confounding from differences in patient health. However, due to the evidence of increased upcoding from vertical integration, Elixhauser comorbidity count could have increased bias instead of reduced bias, which led us to include this specification as a sensitivity analysis instead of as the primary model.^22^ Fourth, we estimated the primary analysis using the unmatched full sample. Finally, we estimated the model including only patients whose admissions were classified as emergent (ie, patients who were less likely to select where they were admitted) in an attempt to further reduce any potential selection bias. All models are reported as percentage point differences to ease interpretation. The primary model is additionally reported as percentage-point change. Statistical significance was determined with a Bonferroni-corrected 2-sided significance threshold of *P* < .01 based on the 7 different outcomes. Analyses were conducted using Stata version 18.0 (StataCorp LLC).

## Results

### Cardiopulmonary Admission Characteristics

After matching, we identified 1 057 715 cardiopulmonary admissions in 1654 hospitals, of which 234 587 were partially integrated and 118 017 were fully integrated admissions (Table 1; eTable 3 in Supplement 1). After propensity score matching, all 3 admission groups were similar in mean (SD) age (nonintegrated, 78.0 [9.9] years; partially integrated, 78.0 [9.9], years, and fully integrated, 78.0 [9.8] years), sex (nonintegrated: female, 48.8% and male, 51.2%; partially integrated: female, 48.9% and male, 51.1%; and fully integrated, female, 48.9% and male, 51.1%), dual-eligibility status (nonintegrated fully dual, 9.5%; partially integrated fully dual, 9.4%; fully integrated fully dual, 9.4%), and Elixhauser comorbidity count (nonintegrated, 5.8; partially integrated, 5.8; and fully integrated, 5.8). All 3 admission types had a similar distribution of cardiopulmonary condition type, with the most common being heart failure, followed by acute myocardial infarction and then pneumonia.

**Table 1.** Demographic Characteristics by Level of Vertical Integration Exposure (N = 1 057 715)^a^

Variable	Participants, No. (%)		
	Nonintegrated (n = 705 111)	Partially integrated (n = 234 587)	Fully integrated (n = 118 017)
Age, mean (SD), y	78.0 (9.9)	78.0 (9.9)	78.0 (9.8)
Race and ethnicity^b^			
Asian	3045 (0.4)	1001 (0.4)	666 (0.5)
Hispanic	5338 (0.8)	1590 (0.8)	905 (0.8)
North American Native	883 (0.1)	290 (0.1)	187 (0.2)
Non-Hispanic Black	64 177 (9.1)	21 328 (9.1)	10 769 (9.1)
Non-Hispanic White	621 783 (88.2)	207 144 (88.3)	103 761 (87.9)
Other^c^	4897 (0.7)	1730 (0.7)	943 (0.8)
Unknown	4988 (0.7)	1504 (0.6)	786 (0.7)
Sex			
Female	343 810 (48.8)	114 807 (48.9)	57 737 (48.9)
Male	351 301 (51.2)	119 780 (51.1)	60 280 (51.1)
Dual eligible			
Nondual	598 456 (84.9)	199 494 (85.0)	100 130 (84.9)
Partially dual	39 562 (5.6)	13 152 (5.6)	6762 (5.7)
Fully dual	67 093 (9.5)	21 941 (9.4)	11 125 (9.4)
Condition			
Acute myocardial infarction	168 098 (23.8)	55 875 (23.8)	27 851 (23.6)
Heart failure	393 290 (55.8)	130 812 (55.8)	66 228 (56.1)
Pneumonia	143 723 (20.4)	47 900 (20.4)	23 938 (20.3)
Elixhauser comorbidity count, mean (SD)	5.8 (2.2)	5.8 (2.2)	5.8 (2.3)

^a^ Nonintegrated admissions were patients not enrolled in a hospital-owned Medicare Advantage plan, partially integrated admissions were patients enrolled in a hospital-owned Medicare Advantage plan admitted at a nonaffiliated hospital, and fully integrated admissions were patients enrolled in a hospital-owned Medicare Advantage plan admitted at the hospital that owns the plan.

^b^ Race and ethnicity were determined according to self-report from Medicare Advantage database.

^c^ No further breakdown is available for this classification.

### Outcomes

Compared with nonintegrated admissions, fully integrated admissions had significantly shorter lengths of stay (adjusted difference, −0.24 days; 95% CI, −0.31 to −0.18 days or −4.3%; 95% CI, −5.6% to −3.3%; *P* < .001); less ICU use (−1.17 percentage points; 95% CI, −1.82 to −0.52 percentage points or −2.9%; 95% CI, −4.5% to −1.3%; *P* < .001); and lower rates of inpatient mortality (−0.61 percentage points; 95% CI, −0.88 to −0.34 percentage points or −11.0%; 95% CI, −16.0% to −6.0%; *P* < .001), 30-day postdischarge mortality (−0.87 percentage points; 95% CI, −1.30 to −0.44 percentage points or −6.3%; 95% CI, −9.5% to −3.2%; *P* < .001), and 30-day postdischarge readmission (−0.78 percentage points; 95% CI, −1.30 to −0.26 percentage points or −4.1%; 95% CI, −6.9% to −1.4%; *P* < .01) (Table 2). Results between fully integrated and nonintegrated admissions were generally consistent when subgrouping by cardiopulmonary condition type, except ICU use and 30-day postdischarge readmission were not significantly different for acute myocardial infarction admissions, and only length of stay was significantly shorter for pneumonia admissions (eTables 4-6 in Supplement 1).

**Table 2.** Adjusted Outcomes by Level of Hospital–Medicare Advantage Vertical Integration Exposure^a^

Outcome	Coefficient (95% CI)			
	Fully integrated vs nonintegrated		Fully integrated vs partially integrated	
	Adjusted marginal effect	Relative difference, %	Adjusted marginal effect	Relative difference, %
Care intensity				
Length of stay	−0.24 (−0.31 to −0.18)^b^	−4.3 (−5.6 to −3.3)^b^	−0.28 (−0.37 to −0.18)^b^	−5.0 (−6.7 to −3.2)^b^
Any ICU use	−1.17 (−1.82 to −0.52)^b^	−2.9 (−4.5 to −1.3)^b^	−1.42 (−2.44 to −0.40)^c^	−3.6 (−6.2 to −1.0)^c^
Clinical				
Inpatient mortality	−0.61 (−0.88 to −0.34)^b^	−11.0 (−16.0 to −6.0)^b^	−0.50 (−0.91 to −0.10)	−8.5 (−15.8 to −1.6)
30-d Postdischarge mortality	−0.87 (−1.30 to −0.44)^b^	−6.3 (−9.5 to −3.2)^b^	−0.74 (−1.37 to −0.12)	−5.3 (−9.9 to −0.8%)
30-d Postdischarge readmission	−0.78 (−1.30 to −0.26)^c^	−4.1 (−6.9 to −1.4)^c^	−0.004 (−0.87 to 0.86)	−0.02 (−4.4 to 4.5)

^a^ All analyses used generalized linear models (Poisson for count outcomes and logit for binary outcomes) adjusted for age, race, sex, dual-eligibility status, and cardiopulmonary condition type with fixed effects for hospital, patient zip code, year, and diagnosis-related group. SEs are clustered at the hospital level. Estimates are marginal effects. Binary outcomes (mortality, readmission, and any intensive care unit [ICU] use) were multiplied by 100 to obtain percentage point differences. Adjusted group means were calculated using the margins command after the adjusted regression in Stata.

^b^ Bonferroni-corrected *P* < .001.

^c^ Bonferroni-corrected *P* < .01.

There was no significant difference in inpatient mortality, 30-day postdischarge mortality, or 30-day postdischarge readmission between fully integrated and partially integrated admissions. Compared with partially integrated admissions, fully integrated admissions had significantly shorter lengths of stay (−0.28 days; 95% CI, −0.37 to −0.18 days or −5.0%; 95% CI, −6.7% to −3.2%; *P* < .001) and less ICU use (−1.42 percentage points; 95% CI, −2.44 to −0.40 percentage points or −3.6%; 95% CI, −6.2% to −1.0%; *P* < .01). Results between fully integrated and partially integrated admissions when subgrouping by cardiopulmonary condition type were similar for length of stay. ICU use was not significantly different between fully integrated and partially integrated admissions when subgrouping by cardiopulmonary condition type.

### Sensitivity Analyses

Our findings were consistent when modeled with linear regression instead of GLMs (eTable 7 in Supplement 1). After adjusting for Elixhauser comorbidity count, results were similar, except 30-day postdischarge mortality and readmission rates were not significantly different than those for nonintegrated admissions (eTable 8 in Supplement 1). Using the full unmatched sample and emergency admissions only, the results were consistent with the matched sample results (eTables 9 and 10 in Supplement 1).

## Discussion

This cross-sectional study found patients with acute myocardial infarction, heart failure, or pneumonia admitted to a hospital that was vertically integrated with a patient’s MA contract had shorter lengths of stay; less ICU use; and lower rates of inpatient mortality, 30-day postdischarge mortality, and 30-day postdischarge readmission compared with patients admitted with a nonintegrated MA contract. In addition, patients with fully integrated admissions had shorter lengths of stay and less ICU use compared with those with partially integrated admissions.

We found that outcomes were better in hospitals that owned an MA contract for those enrolled in the hospital-owned MA contract. This could be due to these hospitals providing better care for patients with integrated admissions than for their peers with nonintegrated admissions. The mechanisms by which this might occur are unknown and warrant further work. It is possible that these patients are admitted to the hospital at slightly less acutely severe stages of disease; for example, patients being monitored in a heart failure disease management program could be sent to the hospital at an early sign of weight gain and congestion rather than waiting for further clinical deterioration. It is also possible that patients’ conditions are managed better due to a more complete set of information being available regarding their medication regimens and previous outpatient testing or encounters.

Our findings should be interpreted in the context of broader literature around vertical integration. Previous studies have also suggested that hospital-owned MA contracts may provide more coordinated care, leading to better patient experiences and health outcomes and less care use, but only when patients are admitted at the hospital that owns the MA contract.^12,22,32^ Likewise, enrollees in older hospital-owned MA contracts report better care experiences than those in newer hospital-owned MA contracts, possibly reflecting a learning curve as hospitals adapt to managing care as insurers.^32^ Additionally, the percentage-point differences tended to be small, especially for care intensity, with less than a 5% relative difference. For length of stay, this is only one-quarter of a day, which is unlikely to be clinically or economically meaningful. However, a 2.9% reduction in ICU use may be more likely to be clinically and economically meaningful. While there was no significant difference when compared with partially integrated admissions, fully integrated admissions were associated with clinical outcomes with adjusted differences likely to be clinically meaningful compared with nonintegrated admissions. Our study builds on prior work that focused on the broader MA population by examining a specific set of beneficiaries admitted for conditions that are common, highly morbid, and the focus of national efforts for care quality improvement, in addition to expanding the study cohort beyond admissions in 2015.^12,14,35^

### Limitations

Although we matched groups by observable characteristics and adjusted for observed patient demographics and hospital, geographic, year, and diagnosis-related group fixed effects, our study may still be limited by potential unobserved differences between patients that may have biased our results. However, any potential selection into hospital-owned MA plans would not bias the comparison between fully integrated and partially integrated admissions. In this comparison, we did not detect differences in clinical outcomes, but we observed reduced care use in fully integrated admissions. This finding suggests that vertical integration allows further use management when beneficiaries are admitted to the hospital that owns their MA contract. Additionally, results were consistent when limiting the sample to those who were admitted emergently and were less likely to select where they were admitted. While the hospital-owned MA contract data are longitudinal, they are not suitable for quasi-experimental methods, such as difference-in-differences estimation, that could further reduce potential bias because the hospital-owned MA contracts were created from the vertical integration. This means that there is no switching from nonintegrated to integrated for the MA contracts, as they are hospital owned from inception, which reduces the ability to use difference in differences. Future research should attempt to address this limitation in an effort to use quasi-experimental methods if possible. Additionally, future research should expand the time period when data become available to estimate the long-term effects of hospital-MA vertical integration.

## Conclusions

In this study, hospital-MA vertical integration was associated with shorter lengths of stay, less ICU use, and lower rates of mortality and readmission among patients admitted for acute myocardial infarction, heart failure, or pneumonia. These findings suggest potential benefits from aligning incentives between insurers and practitioners.

## Notes

Supplement 1. **eTable 1.** *ICD-10* Codes Used to Identify Cardiopulmonary Conditions **eTable 2.** Overdispersion Ratio of Poisson Regression in Primary Analysis **eTable 3.** Years of Hospital Vertical Integration (n = 1654) **eTable 4.** Adjusted Patient and Care Intensity Outcomes by Level of Vertical Integration Exposure for Acute Myocardial Infarction Admissions **eTable 5.** Adjusted Patient and Care Intensity Outcomes by Level of Vertical Integration Exposure for Heart Failure Admissions **eTable 6.** Adjusted Patient and Care Intensity Outcomes by Level of Vertical Integration Exposure for Pneumonia Admissions **eTable 7.** Adjusted Outcomes by Level of Hospital-MA Vertical Integration Exposure Using Linear Regression **eTable 8.** Adjusted Outcomes by Level of Hospital-MA Vertical Integration Exposure With Elixhauser Comorbidity Adjustment **eTable 9.** Adjusted Outcomes by Level of Hospital-MA Vertical Integration Exposure With Full Unmatched Sample **eTable 10.** Adjusted Outcomes by Level of Hospital-MA Vertical Integration Exposure for Emergency Admissions Only **eFigure.** Schematic of Propensity Score–Matching Approach

Supplement 2.
Data Sharing Statement
