# Supplementary material for: Hospital–Medicare Advantage Vertical Integration and Cardiopulmonary Care in Integrated Hospitals
Source: JAMA Health Forum. 2025 Dec 26;6(12):e255648. doi: 10.1001/jamahealthforum.2025.5648 (PMC12743279; doi:10.1001/jamahealthforum.2025.5648)
Supplement: Supplement 1. — eTable 1. ICD-10 Codes Used to Identify Cardiopulmonary Conditions eTable 2. Overdispersion Ratio of Poisson Regression in Primary Analysis eTable 3. Years of Hospital Vertical Integration (n = 1654) eTable 4. Adjusted Patient and Care Intensity Outcomes by Level of Vertical Integration Exposure for Acute Myocardial Infarction Admissions eTable 5. Adjusted Patient and Care Intensity Outcomes by Level of Vertical Integration Exposure for Heart Failure Admissions eTable 6. Adjusted Patient and Care Intensity Outcomes by Level of Vertical Integration Exposure for Pneumonia Admissions eTable 7. Adjusted Outcomes by Level of Hospital-MA Vertical Integration Exposure Using Linear Regression eTable 8. Adjusted Outcomes by Level of Hospital-MA Vertical Integration Exposure With Elixhauser Comorbidity Adjustment eTable 9. Adjusted Outcomes by Level of Hospital-MA Vertical Integration Exposure With Full Unmatched Sample eTable 10. Adjusted Outcomes by Level of Hospital-MA Vertical Integration Exposure for Emergency Admissions Only eFigure. Schematic of Propensity Score–Matching Approach [file jamahealthforum-e255648-s001.pdf]

Supplemental Online Content

Bejarano G, Dixit MN, Philips AP, et al. Hospital-Medicare Advantage vertical integration and cardiopulmonary care in integrated hospitals. *JAMA Health Forum*. 2025;6(12):e255648. doi:10.1001/jamahealthforum.2025.5648

eTable 1. *ICD-10* codes used to identify cardiopulmonary conditions

eTable 2. Overdispersion ratio of Poisson regression in primary analysis

eTable 3. Years of hospital vertical integration (n = 1,654)

eTable 4. Adjusted patient and care intensity outcomes by level of vertical integration exposure for acute myocardial infarction admissions

eTable 5. Adjusted patient and care intensity outcomes by level of vertical integration exposure for heart failure admissions

eTable 6. Adjusted patient and care intensity outcomes by level of vertical integration exposure for pneumonia admissions

eTable 7. Adjusted outcomes by level of hospital-MA vertical integration exposure using linear regression

eTable 8. Adjusted outcomes by level of hospital-MA vertical integration exposure with Elixhauser comorbidity adjustment

eTable 9. Adjusted outcomes by level of hospital-MA vertical integration exposure with full unmatched sample

eTable 10. Adjusted outcomes by level of hospital-MA vertical integration exposure for emergency admissions only

eFigure 1. Schematic of propensity score matching approach

© 2025 Bejarano G et al. *JAMA Health Forum*.

eTable 1: ICD-10 codes used to identify cardiopulmonary conditions

Procedure	ICD-10 Codes
Acute Myocardial Infarction	I2101, I2102, I2109, I2111, I2119, I2121, I2129, I213, I214, I219, I21A1, I21A9
Heart Failure	I110, I130, I132, I501, I509, I5020, I5021, I5022, I5023, I5030, I5031, I5032, I5033, I5040, I5041, I5042, I5043
Pneumonia	J120, J121, J122, J123, J1281, J1289, J129, J13, J14, J150, J151, J1520, J15211, J15212, J1529, J153, J154, J155, J156, J157, J158, J159, J160, J180, J181, J182, J188, J189

© 2025 Bejarano G et al. *JAMA Health Forum*.

eTable 2: Overdispersion ratio of Poisson regression in primary analysis

**Outcome**	**Fully-Integrated vs. Non-Integrated, Overdispersion Ratio**	**Fully-Integrated vs. Partially-Integrated, Overdispersion Ratio**
Length of Stay	3.47	3.24

Notes: All analyses used quasi-Poisson for count outcomes adjusted for age, race, sex, dual status. cardiopulmonary condition type, and hospital, and patient county fixed effects. Standard errors are clustered at the hospital level. Overdispersion ratio greater than 1 is likely to represent overdispersion.

© 2025 Bejarano G et al. *JAMA Health Forum*.

eTable 3: Years of hospital vertical integration (n=1,654)

**Year of vertical integration**	**Count (%)**
1980	35 (2.12%)
1985	45 (4.84%)
1986	8 (0.48%)
1994	10 (0.60%)
1995	19 (1.15%)
1996	162 (9.79%)
1997	101 (6.11%)
1998	5 (0.30%)
1999	64 (3.87%)
2001	29 (1.75%)
2002	19 (1.15%)
2003	21 (1.27%)
2005	73 (4.41%)
2006	85 (5.14%)
2008	141 (8.52%)
2009	44 (2.66%)
2010	11 (0.67%)
2012	33 (2.00%)
2013	72 (4.35%)
2014	30 (1.81%)
2015	274 (16.57%)
2016	31 (1.87%)
2017	25 (1.51%)
2018	30 (1.81%)
2019	38 (2.30%)
2020	50 (3.02%)
2021	61 (3.69%)
2022	105 (6.35%)
2023	26 (1.57%)
2024	7 (0.42%)

© 2025 Bejarano G et al. *JAMA Health Forum*.

eTable 4: Adjusted Patient and Care Intensity Outcomes by Level of Vertical Integration Exposure for Acute Myocardial Infarction Admissions

**Outcome**	**Fully-Integrated vs. Non-Integrated, Coefficient (95% CI)**	**Fully-Integrated vs. Partially-Integrated, Coefficient (95% CI)**
	**Adjusted Marginal Effect**	**Adjusted Marginal Effect**
Patient Outcomes		
Inpatient Mortality	-1.38 (-2.37 to -0.38)**	-0.32 (-1.29 to 1.93)
30 Day Post-Discharge Mortality	-1.24 (-2.20 to -0.28)*	-0.92 (-2.49 to 0.65)
30 Day Post-Discharge Readmission	-0.58 (-1.74 to 0.57)	-0.17 (-1.91 to 1.57)
Care Intensity		
Length of Stay	-0.0004 (-0.09 to 0.09)	-0.21 (-0.35 to -0.07)*
Any ICU Utilization	-1.12 (-2.25 to 0.009)	-2.07 (-3.74 to -0.40)

Notes: Bonferroni corrected p-values where * indicates $p<0.01$ and ** indicates $p<0.001$. All analyses used generalized linear models (Poisson for count outcomes and logit for binary outcomes) adjusted for age, race, sex, and dual status with fixed effects for hospital, patient zip code, year, and diagnostic related group. Standard errors are clustered at the hospital level. Estimates are marginal effects. Binary outcomes (mortality, readmission, any ICU utilization) were multiplied by 100 to get percentage point differences.

© 2025 Bejarano G et al. *JAMA Health Forum*.

eTable 5: Adjusted Patient and Care Intensity Outcomes by Level of Vertical Integration Exposure for Heart Failure Admissions

**Outcome**	**Fully-Integrated vs. Non-Integrated, Coefficient (95% CI)**	**Fully-Integrated vs. Partially-Integrated, Coefficient (95% CI)**
	**Adjusted Marginal Effect**	**Adjusted Marginal Effect**
Patient Outcomes		
Inpatient Mortality	-0.56 (-0.96 to -0.16)*	-1.05 (-1.62 to -0.47)**
30 Day Post-Discharge Mortality	-0.78 (-1.37 to -0.19)**	-0.96 (-1.84 to -0.07)
30 Day Post-Discharge Readmission	-0.90 (-1.50 to -0.29)*	0.11 (-1.09 to 1.30)
Care Intensity		
Length of Stay	-0.16 (-0.25 to -0.07)**	-0.23 (-0.37 to -0.09)**
Any ICU Utilization	-1.04 (-1.77 to -0.30)*	-1.33 (-2.46 to -0.19)

Notes: Bonferroni corrected p-values where * indicates $p<0.01$ and ** indicates $p<0.001$. All analyses used generalized linear models (Poisson for count outcomes and logit for binary outcomes) adjusted for age, race, sex, and dual status with fixed effects for hospital, patient zip code, year, and diagnostic related group. Standard errors are clustered at the hospital level. Estimates are marginal effects. Binary outcomes (mortality, readmission, any ICU utilization) were multiplied by 100 to get percentage point differences.

© 2025 Bejarano G et al. *JAMA Health Forum*.

eTable 6: Adjusted Patient and Care Intensity Outcomes by Level of Vertical Integration Exposure for Pneumonia Admissions

**Outcome**	**Fully-Integrated vs. Non-Integrated, Coefficient (95% CI)**	**Fully-Integrated vs. Partially-Integrated, Coefficient (95% CI)**
	**Adjusted Marginal Effect**	**Adjusted Marginal Effect**
Patient Outcomes		
Inpatient Mortality	-0.62 (-1.94 to 0.70)	0.26 (-1.89 to 2.42)
30 Day Post-Discharge Mortality	-0.76 (-2.02 to 0.51)	0.09 (-2.19 to 2.37)
30 Day Post-Discharge Readmission	-0.85 (-2.15 to 0.44)	-0.83 (-2.57 to 0.90)
Care Intensity		
Length of Stay	-0.14 (-0.25 to -0.03)*	-0.23 (-0.40 to -0.06)*
Any ICU Utilization	-1.33 (-2.61 to -0.05)	-1.05 (-3.45 to 1.34)

Notes: Bonferroni corrected p-values where * indicates $p<0.01$ and ** indicates $p<0.001$. All analyses used generalized linear models (Poisson for count outcomes and logit for binary outcomes) adjusted for age, race, sex, and dual status with fixed effects for hospital, patient zip code, year, and diagnostic related group. Standard errors are clustered at the hospital level. Estimates are marginal effects. Binary outcomes (mortality, readmission, any ICU utilization) were multiplied by 100 to get percentage point differences.

© 2025 Bejarano G et al. *JAMA Health Forum*.

eTable 7: Adjusted Outcomes by Level of hospital-MA Vertical Integration Exposure using linear regression

**Outcome**	**Fully-Integrated vs. Non-Integrated, Coefficient (95% CI)**	**Fully-Integrated vs. Partially-Integrated, Coefficient (95% CI)**
	**Adjusted Marginal Effect**	**Adjusted Marginal Effect**
Patient Outcomes		
Inpatient Mortality	-0.42 (-0.64 to -0.20)**	-0.44 (-0.76 to -0.12)*
30 Day Post-Discharge Mortality	-0.70 (-1.11 to -0.30)**	-0.65 (-1.23 to -0.08)
30 Day Post-Discharge Readmission	-0.76 (-1.27 to -0.25)*	-0.05 (-0.86 to 0.77)
Care Intensity		
Length of Stay	-0.25 (-0.32 to -0.19)**	-0.29 (-0.39 to -0.19)**
Any ICU Utilization	-1.10 (-1.72 to -0.48)**	-1.40 (-2.37 to -0.44)*

Notes: Bonferroni corrected p-values where * indicates $p<0.01$ and ** indicates $p<0.001$. All analyses used linear regression adjusted for age, race, sex, and dual status with fixed effects for hospital, patient zip code, year, and diagnostic related group. Standard errors are clustered at the hospital level. Binary outcomes (mortality, readmission, any ICU utilization) were multiplied by 100 to get percentage point differences.

© 2025 Bejarano G et al. *JAMA Health Forum*.

eTable 8: Adjusted Outcomes by Level of hospital-MA Vertical Integration Exposure with Elixhauser comorbidity adjustment

**Outcome**	**Fully-Integrated vs. Non-Integrated, Coefficient (95% CI)**	**Fully-Integrated vs. Partially-Integrated, Coefficient (95% CI)**
	**Adjusted Marginal Effect**	**Adjusted Marginal Effect**
Patient Outcomes		
Inpatient Mortality	-0.44 (-0.73 to -0.15)*	-0.42 (-0.85 to -0.0008)
30 Day Post-Discharge Mortality	-0.46 (-0.91 to -0.02)	-0.51 (-1.13 to 0.11)
30 Day Post-Discharge Readmission	-0.49 (-1.01 to 0.04)	0.15 (-0.71 to 1.02)
Care Intensity		
Length of Stay	-0.13 (-0.20 to -0.07)**	-0.22 (-0.31 to -0.13)**
Any ICU Utilization	-0.90 (-1.56 to -0.25)*	-1.28 (-2.31 to -0.25)*

Notes: Bonferroni corrected p-values where * indicates p<0.01 and ** indicates p<0.001. All analyses used generalized linear models (Poisson for count outcomes and logit for binary outcomes) adjusted for age, race, sex, dual status, and Elixhauser comorbidity count with fixed effects for hospital, patient county, and diagnostic related group. Standard errors are clustered at the hospital level. Estimates are marginal effects. Binary outcomes (mortality, readmission, any ICU utilization) were multiplied by 100 to get percentage point differences.

eTable 9: Adjusted Outcomes by Level of hospital-MA Vertical Integration Exposure with full unmatched sample

**Outcome**	**Fully-Integrated vs. Non-Integrated, Coefficient (95% CI)**	**Fully-Integrated vs. Partially-Integrated, Coefficient (95% CI)**
	**Adjusted Marginal Effect**	**Adjusted Marginal Effect**
Patient Outcomes		
Inpatient Mortality	-0.26 (-0.42 to -0.09)*	-0.22 (-0.51 to 0.08)
30 Day Post-Discharge Mortality	-0.37 (-0.63 to -0.11)*	-0.38 (-0.81 to 0.06)
30 Day Post-Discharge Readmission	-0.41 (-0.81 to -0.01)	0.07 (-0.63 to 0.78)
Care Intensity		
Length of Stay	-0.14 (-0.20 to -0.09)**	-0.22 (-0.31 to -0.13)**
Any ICU Utilization	-0.79 (-1.26 to -0.33)**	-1.03 (-1.95 to -0.11)

Notes: Bonferroni corrected p-values where * indicates p<0.01 and ** indicates p<0.001. All analyses used generalized linear models (Poisson for count outcomes and logit for binary outcomes) adjusted for age, race, sex, and dual status with fixed effects for hospital, patient county, and diagnostic related group. Standard errors are clustered at the hospital level. Estimates are marginal effects. Binary outcomes (mortality, readmission, any ICU utilization) were multiplied by 100 to get percentage point differences.

eTable 10: Adjusted Outcomes by Level of hospital-MA Vertical Integration Exposure for emergency admissions only

**Outcome**	**Fully-Integrated vs. Non-Integrated, Coefficient (95% CI)**	**Fully-Integrated vs. Partially-Integrated, Coefficient (95% CI)**
	**Adjusted Marginal Effect**	**Adjusted Marginal Effect**
Patient Outcomes		
Inpatient Mortality	-0.47 (-0.71 to -0.22)**	-0.48 (-0.85 to -0.11)*
30 Day Post-Discharge Mortality	-0.79 (-1.24 to -0.35)**	-0.72 (-1.37 to -0.07)
30 Day Post-Discharge Readmission	-0.68 (-1.21 to -0.15)*	-0.06 (-0.93 to 0.80)
Care Intensity		
Length of Stay	-0.25 (-0.32 to -0.18)**	-0.25 (-0.37 to -0.14)**
Any ICU Utilization	-1.24 (-1.89 to -0.59)**	-1.55 (-2.56 to -0.55)*

Notes: Bonferroni corrected p-values where * indicates p<0.01 and ** indicates p<0.001. All analyses used generalized linear models (Poisson for count outcomes and logit for binary outcomes) adjusted for age, race, sex, and dual status with fixed effects for hospital, patient county, and diagnostic related group. Standard errors are clustered at the hospital level. Estimates are marginal effects. Binary outcomes (mortality, readmission, any ICU utilization) were multiplied by 100 to get percentage point differences.

© 2025 Bejarano G et al. *JAMA Health Forum*.

eFigure 1: Schematic of propensity score matching approach

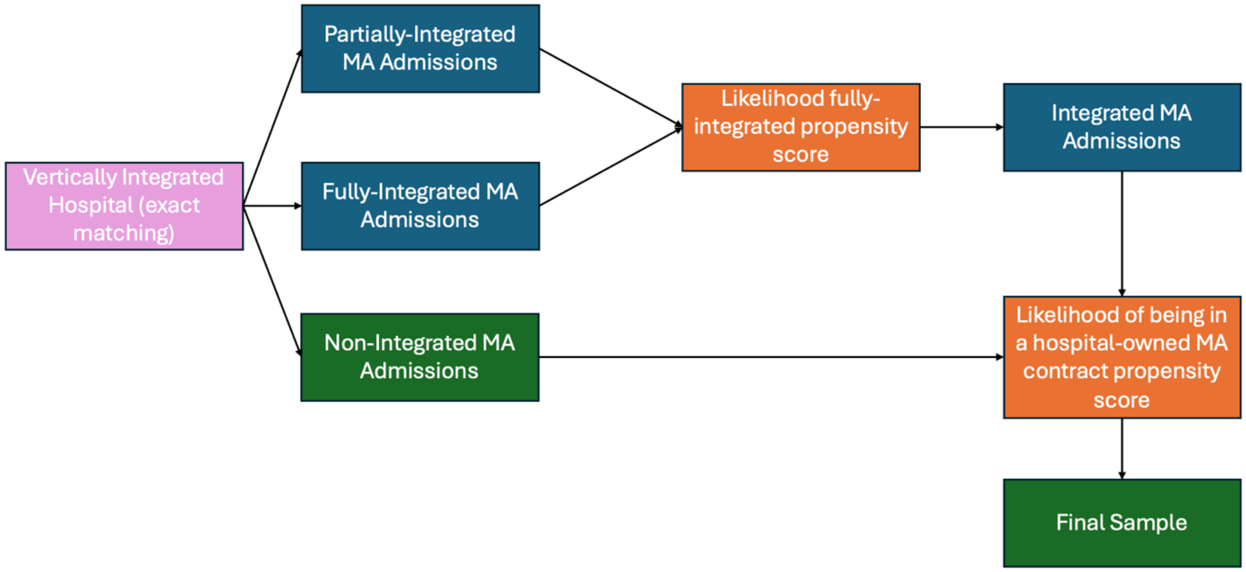

© 2025 Bejarano G et al. *JAMA Health Forum*.
