# Supplementary material for: Hospital–Medicare Advantage Vertical Integration and Cardiopulmonary Care in Integrated Hospitals
Source: JAMA Health Forum. 2025 Dec 26;6(12):e255648. doi: 10.1001/jamahealthforum.2025.5648 (PMC12743279; doi:10.1001/jamahealthforum.2025.5648)
Supplement: Supplement 2. — Data Sharing Statement [file jamahealthforum-e255648-s002.pdf]

# Data Sharing Statement

Bejarano. Hospital–Medicare Advantage Vertical Integration and Cardiopulmonary Care in Integrated Hospitals. *JAMA Health Forum*. Published December 26, 2025. doi:10.1001/jamahealthforum.2025.5648

**Data**
**Data available:** No

**Additional Information**
**Explanation for why data not available:** The data used in this study is under a data use agreement with CMS and therefore cannot be made available. The code used in this study can be requested from the corresponding author.
